# Reduced mitochondrial respiration in T cells of patients with major depressive disorder

**DOI:** 10.1016/j.isci.2021.103312

**Published:** 2021-10-16

**Authors:** Stefanie Gamradt, Helge Hasselmann, Aline Taenzer, Jelena Brasanac, Victoria Stiglbauer, Arne Sattler, Max Sajitz-Hermstein, Sylwia Kierszniowska, Caren Ramien, Jan Nowacki, Lea Mascarell-Maricic, Katja Wingenfeld, Dominique Piber, Andreas Ströhle, Katja Kotsch, Friedemann Paul, Christian Otte, Stefan M. Gold

**Affiliations:** 1 Charité – Universitätsmedizin Berlin, Klinik für Psychiatrie und Psychotherapie, Campus Benjamin Franklin, Hindenburgdamm 30, 12203 Berlin, Germany; 2 Institut für Neuroimmunologie und Multiple Sklerose (INIMS), Zentrum für Molekulare Neurobiologie, Universitätsklinikum Hamburg Eppendorf, 20251 Hamburg, Germany; 3 Charité – Universitätsmedizin Berlin and Max Delbrueck Center for Molecular Medicine, NeuroCure Clinical Research Center (NCRC), Campus Mitte, 10117 Berlin, Germany; 4 Charité – Universitätsmedizin Berlin, Medizinische Klinik m.S. Psychosomatik, Campus Benjamin Franklin, 12203 Berlin, Germany; 5 Charité – Universitätsmedizin Berlin, Klinik für Allgemein- und Viszeralchirurgie, Campus Benjamin Franklin, 12203 Berlin, Germany; 6 metaSysX GmbH, 14476 Potsdam, Germany; 7 Charité – Universitätsmedizin Berlin, Klinik für Psychiatrie und Psychotherapie, Campus Mitte, 10117 Berlin, Germany

**Keywords:** Cellular neuroscience, Immunology, Metabolomics

## Abstract

Converging evidence indicates that major depressive disorder (MDD) and metabolic disorders might be mediated by shared (patho)biological pathways. However, the converging cellular and molecular signatures remain unknown. Here, we investigated metabolic dysfunction on a systemic, cellular, and molecular level in unmedicated patients with MDD compared with matched healthy controls (HC). Despite comparable BMI scores and absence of cardiometabolic disease, patients with MDD presented with significant dyslipidemia. On a cellular level, T cells obtained from patients with MDD exhibited reduced respiratory and glycolytic capacity. Gene expression analysis revealed increased carnitine palmitoyltransferase IA (*CPT1a*) levels in T cells, the rate-limiting enzyme for mitochondrial long-chain fatty acid oxidation. Together, our results indicate metabolic dysfunction in unmedicated, non-overweight patients with MDD on a systemic, cellular, and molecular level. This evidence for reduced mitochondrial respiration in T cells of patients with MDD provides translation of previous animal studies regarding a putative role of altered immunometabolism in depression pathobiology.

## Highlights

• MDD patients display signs of metabolic imbalance on a systemic level
• Mitochondrial respiration and glycolysis are decreased in T cells of MDD patients
• Key cellular metabolic markers negatively correlate with depression severity
• Increased expression of CPT1a in T cells correlates with many serum metabolites

## Graphical abstract

**Figure:**
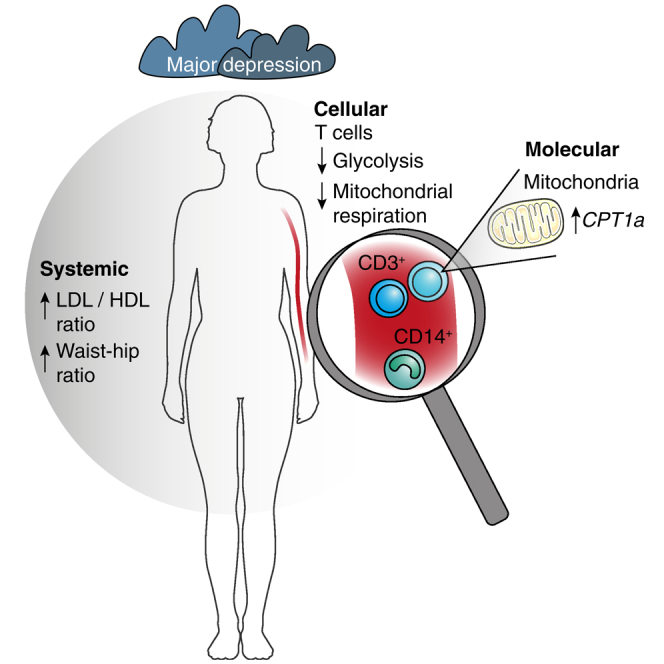

## Introduction

Major depressive disorder (MDD) is a highly prevalent psychiatric disease associated with a substantial disease burden (Whiteford et al., 2013) and increased mortality (Machado et al., 2018; Plana-Ripoll et al., 2019). A large body of literature indicates that MDD is associated with alterations in several (neuro)biological systems, including dysfunction in metabolic pathways (Otte et al., 2016). Indeed, comorbid depression is particularly common in patients with cardiometabolic disorders (Gold et al., 2020) and accumulating evidence from large-scale epidemiological studies has revealed a bidirectional association between MDD and an increased risk for heart disease (Nicholson et al., 2006), diabetes (Mezuk et al., 2008), and metabolic syndrome (Pan et al., 2012). This seems to extend to subclinical metabolic dysregulation, as a metabolomics analysis in 5,283 cases and 10,145 healthy controls revealed evidence for a distinct profile of dyslipidemia in MDD (Bot et al., 2020). These associations were consistent across sex, age, and body mass index (BMI) strata, indicating that they are unlikely to be epiphenomena of medical comorbidities in MDD.

Converging evidence indicates that the link between metabolic dysfunction and MDD could be mediated via common (patho)biological pathways. For example, metabolic diseases and MDD have been shown to share genetic risk factors, as demonstrated by a risk variant overlap between BMI and MDD, particularly in atypical depression (Milaneschi et al., 2017). Of note, these mainly comprise genes that are involved in inflammatory pathways.

Given the importance of inflammation in the pathophysiology of MDD (Miller and Raison, 2016) and the crucial role of metabolic programming for immunity (Buck et al., 2017), it seems biologically plausible that metabolic dysfunction in general, and altered immunometabolism in particular, could contribute to the pathogenesis of depression (Milaneschi et al., 2020). In line with this notion, recent studies using animal models have provided direct evidence for a causal role of metabolic dysfunction in depression: for example, diet- or genetically induced obesity has been shown to induce depression-like behavior in mice (Ogrodnik et al., 2019). Of note, this effect was independent of weight gain but was mediated via inflammatory mechanisms. On a cellular level, in another study, chronic stress caused T cell-specific disturbances of mitochondrial fusion and respiration (Fan et al., 2019). Intriguingly, transfer of these T cells to non-stressed recipient mice was sufficient to cause anxiety/depression-like behavior (Fan et al., 2019).

However, whether these findings can be translated to humans remains unknown. Thus, although an impaired mitochondrial function in a number of tissues and cell populations, including in the central nervous system (CNS) (Pei and Wallace, 2018), may play a role in depression pathobiology, studying these aspects in the immune system, and specifically in T cells, might be particularly relevant. In the present study, we therefore examined metabolic dysfunction in unmedicated patients with MDD (but without metabolic or cardiovascular disease) on a systemic, cellular and molecular level, compared with healthy controls (HC) individually matched for important potential confounders (age, sex, BMI, smoking status).

## Results

An overview of the clinical and demographic characteristics of the study sample is provided in Table 1 (individual patient data are provided in Table S2).

**Table 1:** Sample characteristics

	MDD (n = 28)	HC (n = 28)	Test statistics	p Value^a^
Age, mean years (SD)	32.2 (11.7)	32.2 (10.5)	T(_df=27_) = 0	>.99
BMI, mean kg/m^2^ (SD)	24.5 (3.4)	23.9 (3.4)	T(_df=27_) = 1.32	.20
% Females (n)	75 (21)	75 (21)	Χ^2^(_df=1_) = 0	>.99
% Current smokers (n)	25 (7)	25 (7)	Χ^2^(_df=1_) = 0	>.99
MADRS, mean score (SD)	25.3 (5.4)	0.9 (1.4)	T(_df=27_) = 25.09	<.01
BDI-II, mean score (SD)	30.3 (7.0)	2.3 (3.1)	T(_df=27_) = 17.76	<.01
BAI, mean score (SD)	21.9 (13.2)	3.6 (3.1)	T(_df=27_) = 6.99	<.01
CTQ total score (SD)	40.8 (15.5)	35.1 (11.3)	T(_df=27_) = 1.42	.17
% MDD subtype (n)	64.3 (18)	–		–
% Melancholic (n)	60.7 (17)	–		–
% Atypical (n)	3.7 (1)	–		–

BAI, Beck Anxiety Inventory; BDI-II, Beck Depression Inventory II; BMI, body mass index; CTQ, Childhood Trauma Questionnaire; HC, healthy control; MADRS, Montgomery-Asberg Depression Rating Scale; MDD, major depressive disorder.

Unless specified otherwise, values represent mean (standard deviation).

a Paired-samples t test for continuous and McNemar's test for dichotomous variables.

### Patients with MDD display subtle signs of systemic metabolic dysfunction

**Figure 1:**
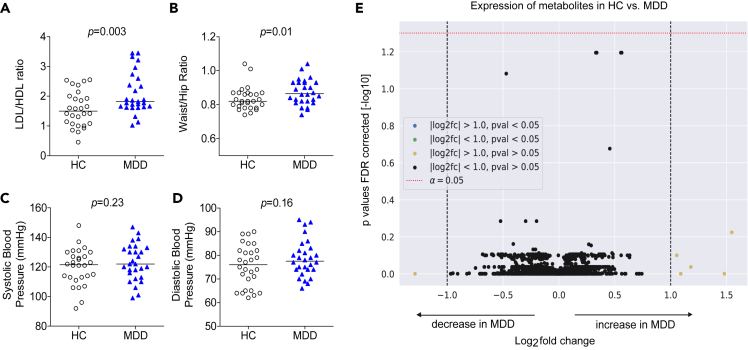
Systemic metabolic imbalance in MDD (A–D) Serum lipids (HDL, high density lipoprotein and LDL, low density lipoprotein, here shown as LDL/HDL ratio) (A), waist/hip ratio (B), as well as systolic (C) and diastolic (D) blood pressure levels were determined in 28 patients with MDD and matched HC. Raw data and group median are shown. Wilcoxon signed-rank test was used to compare groups. (E) Serum metabolomics group comparison in HC versus MDD. In total, 3,511 features were detected by liquid and gas chromatography-mass spectrometry. Dashed lines on the x axis indicate threshold values separating metabolites with >2-fold expression in MDD versus HC (+1) or <0.5-fold expression in MDD versus HC (−1). The dotted line on the y axis represents the threshold value for the selected level of significance (p < 0.05).

Despite close matching for sex, age, BMI, and smoking status, as well as the absence of cardiometabolic disorders in both groups, patients with MDD exhibited subtle signs of metabolic dysfunction compared to healthy controls (HC) (Figure 1). Specifically, patients with MDD showed a higher LDL/HDL ratio (Figure 1A) and a higher waist/hip ratio (WHR) (Figure 1B). Systolic and diastolic blood pressure readings were similar between the groups (Figures 1C and 1D). Group differences in LDL/HDL ratio and WHR remained significant in secondary analyses using Bonferroni correction for multiple testing. Serum metabolites, measured by mass spectrometry-based metabolomics and lipidomics in order to identify further global metabolic signatures in patients with MDD, did not reveal any additional differences between MDD and HC, at least none large enough to be detected with metabolome-wide correction in our sample (Figure 1E).

### Key parameters of mitochondrial respiration and glycolytic activity are reduced in T cells from patients with MDD

**Figure 2:**
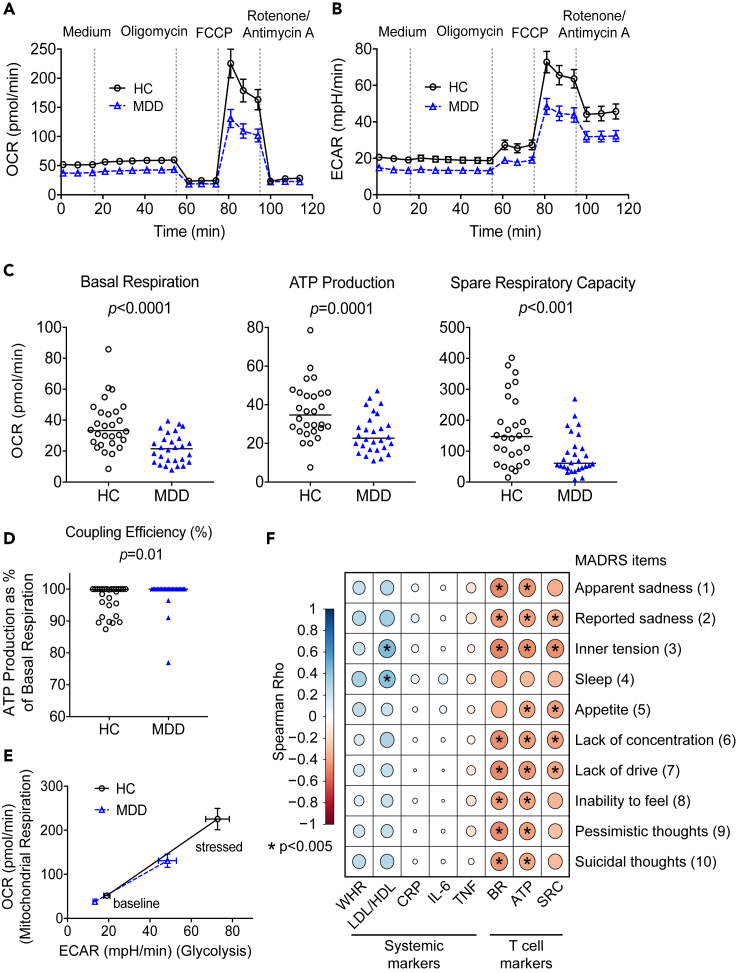
Decreased mitochondrial respiration in T cells of patients with MDD (A and B) To quantify mitochondrial respiration (A) as well as glycolytic activity (B) of CD3^+^ T cells from 28 patients with MDD and 28 matched HC different modulators of respiration were added sequentially to the cells (see also Figure S1) and the oxygen consumption rate (OCR) as well as the extracellular acidification rate (ECAR) were determined over time in a Seahorse XFe96 Analyzer (mean ± SEM is shown, all assays run in three to five replicates for each subject). (C and D) The main parameters of mitochondrial respiration (basal respiration, ATP production, and spare respiratory capacity) (C) as well as the coupling efficiency expressed as the % of basal respiration coupled to ATP production (CE%) (D) are shown. See also Figure S1 for further information regarding the calculation of these parameters from OCR measurements. Individual patient data and median are shown. Wilcoxon signed-rank test was used to compare groups. (E) OCR and ECAR values under baseline and stressed conditions (after addition of Oligomycin and FCCP) were plotted against each other to visualize the energy phenotype of the cells (mean ± SEM shown). (F) Spearman rank correlations of systemic and T cell markers with individual MADRS items. Circle sizes indicate magnitude of correlation, color shading depicts magnitude and direction of correlation (Spearman's rho, see legend). Asterisks were added to the circles if p < 0.005. Oligomycin, ATP synthase inhibitor; carbonyl cyanide-4(trifluoromethoxy) phenylhydrazone (FCCP), uncoupling agent; Rotenone/Antimycin A, complex I/III inhibitors; BR, basal respiration; CRP, C-reactive protein; IL-6, interleukin 6; LDL/HDL, low/high density lipoprotein; MADRS, Montgomery-Asberg Depression Rating Scale; SRC, Spare Respiratory Capacity; TNF, tumor necrosis factor; WHR, waist/hip ratio. Individual patient data for the Seahorse readouts presented here are provided in the supplementary data file, Data S1.

On a cellular level, we observed pronounced impairment of major metabolic readouts in MDD. T cells (defined by CD3 positivity) from patients with MDD exhibited lower levels of oxygen consumption (OCR) (Figure 2A) and lower extracellular acidification rates (ECAR) (Figure 2B), indicative of impaired mitochondrial respiration and decreased glycolytic capacity, especially under cellular stress (Figure 2E). In the primary analysis, significant group differences were observed in T cells from patients with MDD showing decreased basal respiration, lower ATP production, and diminished spare respiratory capacity (Figure 2C). Moreover, basal respiration was to a large extent coupled to ATP production with almost no H^+^ (proton) leak (Figure 2D). With the exception of coupling efficiency, all of these markers were also significantly different after correction for multiple comparisons in the secondary analyses. In monocytes (Figure S2) only one readout (coupling efficiency) was significantly different between MDD and controls in the primary analysis, and this difference was no longer significant after correcting for multiple comparisons. Together, these data suggest a cell-specific reduction of mitochondrial respiration in T cells of patients with MDD.

In order to explore the link of these markers with clinical phenotype, we examined associations of systemic and T cell markers with clinician-rated depression severity as measured by the Montgomery-Asberg Depression Rating Scale (MADRS) (Montgomery and Asberg, 1979), with a higher MADRS score indicative of more severe depression (Figure 2F). Among systemic markers, only LDL/HDL ratio showed significant positive correlation with two MADRS items, inner tension and sleep. In contrast, correlations of clinical phenotype were more robust for all cellular markers, where basal respiration, ATP production, and spare respiratory capacity of T cells were significantly negatively correlated with all MADRS items, except for sleep.

### Reduced mitochondrial respiration in patients with MDD is unlikely to be explained by global shifts in T cell phenotype or immune senescence/exhaustion

**Figure 3:**
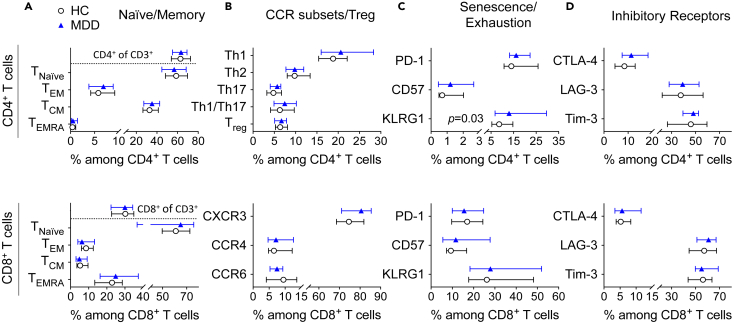
T cell phenotype and senescence/exhaustion markers in MDD (A–D) PBMCs from patients with MDD and matched HC were examined by flow cytometry. CD4^+^ (upper panel) and CD8^+^ T cell subpopulations (lower panel) were analyzed for the expression of markers classifying differentiation (CD45RA, CCR7; n = 25) (A), chemokine receptors (CXCR3, CCR6, CCR4; n = 25) or regulatory T cell markers (CD25, CD127; n = 22) (B), markers indicative of exhaustion or senescence (PD-1, KLRG1, CD57; n = 19–20) (C) or inhibitory receptors (CTLA-4, LAG-3, Tim-3; n = 20) (D). Naive/Memory subpopulations were defined as T_Naive_ (CCR7^+^/CD45RA^+^), T_EM_ (CCR7^−^/CD45RA^−^), T_CM_ (CCR7^+^/CD45RA^−^), and T_EMRA_ (CCR7^−^/CD45RA^+^). T helper cell subsets were defined as Th1 (CXCR3^+^/CCR6^−^), Th2 (CXCR3^−^/CCR6^−^/CCR4^+^), Th17 (CXCR3^−^/CCR6^+^/CCR4^+^), and Th1/Th17 cells (CXCR3^+^/CCR6^+^) (n = 25). For the analysis of inhibitory receptors, PBMCs were stimulated with 10 μg/mL of α-CD3 and 1 μg/mL α-CD28 for 48 h. Median and interquartile ranges are shown. Wilcoxon signed-rank test was used to compare groups. All p values > 0.1 if not otherwise indicated.

Different T cell subpopulations have distinct metabolic profiles, depending on their differentiation stage, activation status, or cellular senescence/exhaustion (Geltink et al., 2018). To rule out the possibility that our observed changes in mitochondrial respiration were merely the result of an over- or underrepresentation of certain T cell subpopulations in patients with MDD, we conducted flow cytometric phenotyping analyses. Among all the phenotypes tested, only one marker (KLRG1) showed significant group differences between MDD and HC in the primary analysis; however, this did not survive adjustment for multiple testing in secondary analyses (Figure 3). Overall, group differences in T cell differentiation or senescence/exhaustion are thus unlikely to explain the pronounced differences in T cell metabolism.

### Decreased mitochondrial respiration in patients with MDD is not secondary to impairments in antiviral immunity

Growing evidence suggests an association between metabolically exhausted T cells and chronic infections. Moreover, prior work has shown that patients with MDD have a higher risk for persistent herpes virus infections like Epstein-Barr virus (EBV) or cytomegalovirus, both before and after onset of depressive symptoms (Andersson et al., 2016). We therefore examined whether the observed reduction in mitochondrial respiration of T cells in patients with MDD could be linked to differences in antiviral immune responses. We chose EBV (pooled EBNA-1, LMP2a and BZLF1 peptide pools) as an exemplary antigen as we expected 90%–95% seropositivity in adults. As control antigen, MP65 from *Candida albicans* was applied, a yeast-like fungus that occurs as a facultative pathogenic commensal on skin and mucosal surfaces and has so far not been linked to the occurrence of MDD. Median frequency of EBV- and MP65-reactive T cells was similar in both groups (4 out of 10,000 CD4^+^ T cells for EBV and 3 out of 10,000 for MP65) (Figure S3). As expected for a recall antigen, CD45RA^−^ T cells, i.e., bona fide memory T cells, made up almost 100% of the EBV-reactive T cell population in both HC and patients with MDD. The same was true for the control antigen MP65. Antigen-specific T cells obtained from both study groups were capable of producing comparable amounts of interferon gamma (IFN-γ) in response to the viral and the fungal stimulus. Moreover, we could not detect any significant differences with regard to the expression of exhaustion markers (PD-1) or markers indicative of cell senescence (CD57) that are typically upregulated in chronic viral infections with continuous antigen exposure. Together, these data suggest that metabolic alterations in MDD are not secondary to group differences in antiviral immunity, at least for common persistent infections such as EBV.

### Cell-specific expression of key metabolic regulators

**Figure 4:**
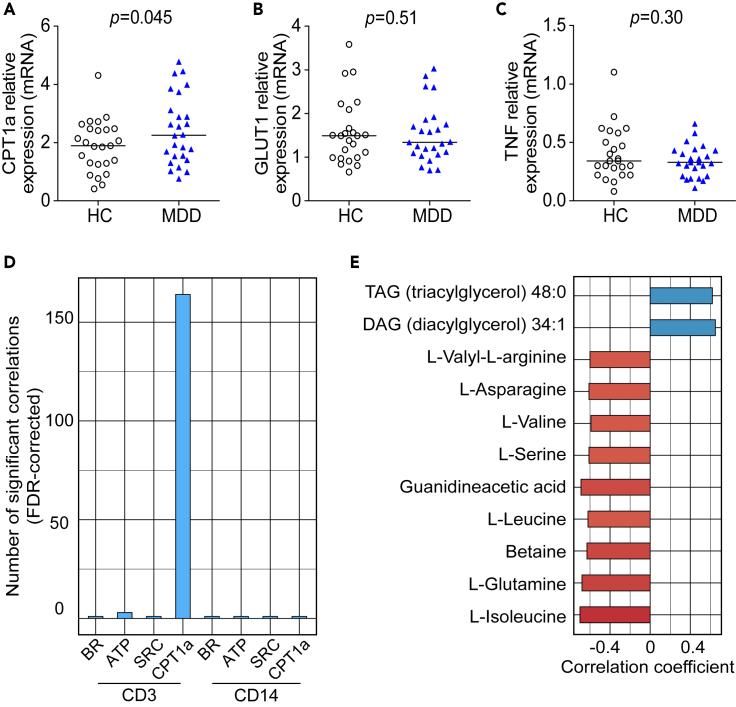
Expression of key regulators of cellular metabolism in T cells of patients with MDD (A–C) mRNA expression levels of *CPT1a* (A), *SLC2A* (encoding GLUT1) (B), and *TNF* (C) in T cells of patients with MDD (n = 25) compared with matched controls (n = 25). Gene expression is shown as fold change relative to housekeeping genes (*TBP* and *IPO8*). Individual patient data and median are shown. Wilcoxon signed-rank test was used to compare groups. (D-E) The numbers of metabolome-wide significant correlations (of 3,511 detected features) are shown for CD3 and CD14 cellular mitochondrial readouts (BR, basal respiration; ATP, ATP production; SR, spare respiratory capacity) (D). Top known metabolites significantly correlated with CD3 *CPT1a* expression (E). CPT1a, carnitine palmitoyltransferase 1 A; GLUT1, glucose transporter 1; TNF, tumor necrosis factor; HC, healthy controls; MDD, major depressive disorder.

Finally, we explored if the transcriptional profile of T cells from patients with MDD indicated metabolic reprogramming. Using targeted cell-specific gene expression analysis by qPCR, we found that T cells of patients with MDD exhibited significantly higher levels of carnitine palmitoyltransferase 1A (*CPT1a*), which encodes a rate-limiting mitochondrial enzyme of long-chain fatty acid (FA) oxidation (Figure 4A). However, expression of *SLC2A1*, encoding the membrane glucose transporter type 1 (GLUT1), the main glucose transporter during T cell activation, and a key inflammatory cytokine (*TNF*) was unaltered in patients with MDD compared with controls (Figures 4B and 4C). This effect was more pronounced in T cells but still detectable in monocytes (Figure S4).

To explore additional shifts, we conducted post hoc analyses of cell-specific expression of key metabolic proteins by Metflow in a subset of n = 5 MDD cases and n = 5 matched healthy controls with sufficient peripheral blood mononuclear cells (PBMCs) available for analysis. The following six key regulators (as markers of different major metabolic pathways) were chosen: CPT1a (key regulator of FA oxidation), GLUT1 (glucose uptake), Glucose-6-phosphate-dehydrogenase (G6PD; oxidative phosphorylation), Hexokinase 1 (HK1; glycolysis), Isocitrate dehydrogenase 2 (IDH2; tricarboxylic acid cycle), and Peroxiredoxin 2 (PRDX2; antioxidant). Our results showed no major differences between MDD and HCs in geometric mean fluorescence intensities of these proteins, at least none that were large enough to be detected in this small subset. Also, no significant correlations between metabolic proteins and CD3 and CD14 Seahorse readouts were found after correction for multiple comparisons (Figure S5).

In order to explore potential correlates of altered cellular metabolism and gene expression, we investigated if CD3 and CD14 cellular readouts correlated with global serum metabolites. Results showed *CPT1a* gene expression in T cells was the only one with substantial correlations, with 164 serum metabolites significantly associated after metabolome-wide correction (Figure 4D), including acylglycerols among the top hits (Figure 4E).

## Discussion

The current study examined metabolic dysfunction in unmedicated and otherwise healthy MDD and HC. Our results showed that MDD is characterized by metabolic dysfunction on a systemic, cellular, and molecular level, even in the absence of overt metabolic disease and when controlling for important confounders.

On a systemic level, we have confirmed findings of a recent hypothesis-free metabolomics study suggesting dyslipidemia in MDD (Bot et al., 2020) that is independent of obesity, sex, age, or other clinical descriptors and may thus be an early manifestation of MDD and potentially involved in its pathobiology. This phenomenon could provide a biological pathway for the significantly elevated risk of cardiometabolic disease in patients with depression.

On a cellular level, we obtained evidence for impaired mitochondrial respiration specifically in T cells of patients with MDD. Our analyses also showed that these alterations are unlikely to simply reflect an epiphenomenon of shifts in T cell differentiation, activation, or senescence/exhaustion. This finding corroborates and extends existing evidence of mitochondrial dysfunction in depression (Allen et al., 2018), which has also been described outside the CNS, e.g., in platelets (Hroudova et al., 2013) and PBMCs (Karabatsiakis et al., 2014; Boeck et al., 2018). Of importance, the metabolic profiles of T cells seen in our MDD cohort bear striking similarity to those reported for T cells obtained from mice after chronic stress (Fan et al., 2019). In this previous study, transfer of these T cells to non-stressed mice in the animal model was sufficient to elicit depression/anxiety-like behavior (Fan et al., 2019), indicating that they could be causally involved in the pathogenesis of depression. Here, our data, obtained with the same readout system for T cell metabolism, provide a translational link from the experimental mouse model to major depression in humans. One intriguing observation here was that the main mitochondrial readouts in T cells (basal respiration, spare respiratory capacity, ATP production) did not show strong correlations with levels of systemic metabolites, using an unbiased metabolomics approach. Although one has to be careful with such interpretations of sequence or cause and effect from purely cross-sectional, observational data, this is in line with the notion that reduced mitochondrial respiration in T cells might be a primary event rather than simply an epiphenomenon. This could have some relevance from a therapeutic perspective; modulating cellular processes in the peripheral immune system, as compared with the CNS, might be easier to achieve.

On a molecular level, T cells from patients with MDD showed increased mRNA levels of *CPT1a*, the rate-limiting enzyme for mitochondrial FA oxidation. *CPT1a* codes for carnitine palmitoyltransferase 1A, which regulates the transport of long-chain FAs across the inner mitochondrial membrane and makes them available for FA oxidation. Therefore, increased levels of *CPT1a* gene expression and unaltered levels of *SLC2A1* (encoding GLUT1) at the same time might be suggestive of an increased reliance on lipolysis and/or ß-oxidation, rather than glycolysis for metabolism; however, we did not measure this directly. The observed correlations of *CPT1a* expression in T cells with many metabolites (including acylglycerols among the top hits) may indicate that *CPT1a* upregulation is more likely an epiphenomenon of systemic metabolic changes in MDD, although we obviously cannot infer cause and effect from our observational data. Ultimately, this finding could add to the emerging evidence that targeting cellular metabolism offers a potential therapeutic pathway in depression: A study in an animal model of depression (Morkholt et al., 2017) has provided evidence that chronic stress increases *CPT1a* expression both in the CNS and in the immune system and that treatment with etomoxir decreases depression-like behavior in rats. Etomoxir has been in clinical development for cardiometabolic disorders (diabetes and heart failure) but was discontinued owing to hepatotoxicity. Moreover, recent evidence has suggested that etomoxir may exert immunomodulatory properties on T cells (Raud et al., 2018), which are independent of CPT1a, so caution is warranted.

A recent metabolomics study in the general population (Zacharias et al., 2021) found decreased serum levels of laurylcarnitine in individuals with elevated levels of depressive symptoms. This metabolite is involved in the transport of (long-chain) FAs from the cytosol to mitochondria for subsequent β-oxidation. Together with our data, this supports the notion of altered FA oxidation and/or mitochondrial activity in depression.

In sum, our study provides evidence for metabolic dysfunction on a systemic, cellular, and molecular level in MDD, extending previous mechanistic insights from animal models and lending further support to the notion of depression as a putative “immunometabolic” disease.

### Limitations of the study

Several limitations of our study should be noted. First, although our analyses were controlled for medical comorbidities, obesity, sex, age, and smoking status, the sample size of our study is relatively small. However, given that our results on systemic metabolic markers closely mirror findings from larger studies of MDD versus HC (i.e., evidence for lower HDL and higher LDL), we are confident that our results in this relatively small sample are generalizable to other MDD patient populations. Nevertheless, our findings need replication in larger, and particularly in longitudinal studies. We have carefully explored alternative explanations for changes in cellular metabolism (including altered immune cell composition, differentiation, senescence/exhaustion, and antiviral T cell immunity), and it appears that altered cellular metabolism can be observed without changes in these parameters.

Although the study groups were matched for sex and age, our sample was predominantly female, as would be expected for MDD. Owing to the low number of males, our study was not powered to test sex differences in mitochondrial respiration. Such analyses should thus be conducted in larger samples to examine generalizability of our findings to both sexes or putative sex differences. For anyone interested in separately analyzing our main readouts by sex, we have provided patient-level information on sex in Table S2 and Data S1.

In order to unravel the implications of reduced mitochondrial respiration in MDD, future studies should directly test different cellular functions putatively driven by altered immunometabolism in MDD. Unfortunately, for the present study, we were limited with regard to the amount of biological material that could be obtained from each participant (e.g., acceptable limits for blood draw volumes as set out in the ethics review and informed consent procedures). Thus, although we conducted extensive experiments to analyze mitochondrial respiration and key metabolic processes and proteins/enzymes as well as relevant control readouts, we did not directly assess potential group differences in a range of additional associated cellular functions. Based on our results, future studies should expand our study by directly probing FA uptake and transport in T cells (e.g., CD36, FA-binding proteins). In addition, FA oxidation assays could provide insights into whether the observed increase in *CPT1a* also reflects a higher ability of T cells for FA oxidation. Moreover, it would be relevant to better understand functional and structural changes within the cells, such as quantification of mitochondrial mass (e.g., by MitoTracker labeling), visualization of mitochondrial fusion and fission, assessment of oxygen consumption in isolated mitochondria, and measurement of reactive oxygen species (e.g., via MitoSOX). Changes in the mitochondrial membrane potential were not measured in our study but could, for example, be estimated by the use of fluorescent dyes like tetramethylrhodamine, methyl ester (TMRM) or 5,5′,6,6′-tetrachloro-1,1′,3,3′-tetraethyl-imidacarbocyanine iodide (JC-1) in future studies, as these markers accumulate in healthy cells with intact mitochondrial membrane potential (Perry et al., 2011). Such measurements might further substantiate findings on reduced mitochondrial respiration (and ATP production in particular) in T cells of patients with MDD as the transmembrane electrochemical gradient is the driver of ATP synthesis.

It should be noted that the mitochondrial alterations we have detected in peripheral immune cells, and in T cells specifically, do not necessarily reflect mitochondrial aberrations in other cells and tissues with relevance for MDD, most notably the CNS. In fact, mitochondrial function is typically regulated in a highly cell- and tissue-specific fashion (Fernández-Vizarra et al., 2011). However, mitochondrial dysfunction in T cells is interesting in its own right owing to their putative role in the pathogenesis of depression-like symptoms as animal models have demonstrated (Fan et al., 2019). Thus, future studies may combine analyses of key mitochondrial parameters in central and peripheral (immune) cells and tissues in animal models of depression to expand our knowledge on the influence of an altered peripheral immunometabolism on the CNS in MDD.

We have previously reported that certain shifts in the T cell compartment can be detected in MDD, at least in treatment-naïve patients without any psychiatric comorbidity, specifically with regard to the expression of chemokine receptors (CXCR3, CCR6) (Patas et al., 2018). We could not replicate this in the present cohort (where some patients, although untreated at the time of study, had previously been treated with antidepressants). Thus, we cannot rule out that there may be subtle shifts in T cell subset compositions (e.g., signs of CD4 T cell exhaustion/senescence as indicated by KLRG1 expression) in MDD. Larger studies are thus needed to explore this further.

Finally, the sequence of metabolic, immunological, and other (neuro)biological substrates of depression remains incompletely understood and should be explored in detail in appropriate animal models and longitudinal clinical studies, including such analyses within the context of randomized controlled trials. For example, we are currently conducting a randomized trial of add-on statins in patients with comorbid MDD and obesity, where cellular and molecular substrates of mitochondrial function are monitored as secondary endpoints (see Otte et al., 2020). This and similar approaches will help to further dissect the nature of the relationship between depression, metabolic dysfunction, and immunometabolism.

## STAR★Methods

### Key resource table

**Table:** 

REAGENT or RESOURCE	SOURCE	IDENTIFIER
**Antibodies**		

Brilliant Violet 421™ anti-human CD3 (OKT3)	Biolegend	Cat# 317344; RRID:AB_2565849
Brilliant Violet 510™ anti-human CD8 (SK1)	Biolegend	Cat# 344732; RRID:AB_2564624
FITC anti-human HLA-DR (LN3)	Biolegend	Cat# 327006; RRID:AB_893569
PE anti-human CD 197 (CCR7) (G043H7)	Biolegend	Cat# 353204; RRID:AB_10913813
PerCPCy5.5 anti human CD4 (RPA-T4)	Biolegend	Cat# 300530; RRID:AB_893322
PE/Cy7 anti human CD45RA (HI100)	Biolegend	Cat# 304126; RRID:AB_10708879
APC anti-human CD38 (HB-7)	Biolegend	Cat# 356606; RRID:AB_2561902
PE anti-human CD183 (CXCR3) (G025H7)	Biolegend	Cat# 353706; RRID: AB_10962912
PE/Cy7 anti-human CD196 (CCR6) (G034E3)	Biolegend	Cat# 353418; RRID:AB_10916518
APC anti-human CD194 (CCR4) (L291H4)	Biolegend	Cat# 359408; RRID:AB_2562429
Brilliant Violet 421™ anti-human FOXP3 (206D)	Biolegend	Cat# 320124; RRID:AB_2565972
Brilliant Violet 510™ anti-human CD3 (SK7)	Biolegend	Cat# 344828; RRID:AB_2563704
PE anti-human CD279 (PD-1) (EH12.2H7)	Biolegend	Cat# 329906; RRID:AB_940483
PerCP/Cyanine5.5 anti-human IFN-γ (4S.B3)	Biolegend	Cat# 502526; RRID:AB_961355
APC anti-human CD57 (HNK-1)	Biolegend	Cat# 359610; RRID:AB_2562757
Brilliant Violet 421™ anti-human CD185 (CXCR5) (J252D4)	Biolegend	Cat# 356920; RRID:AB_2562303
PE anti-human CD25 (M-A251)	Biolegend	Cat# 356104; RRID:AB_2561861
APC anti-human CD127 (IL-7Rα) (A019D5)	Biolegend	Cat# 351316; RRID:AB_10900804
APC/Cyanine7 anti-human CD8a (RPA-T8)	Biolegend	Cat# 301016; RRID:AB_314134
Brilliant Violet 421™ anti-human CD366 (Tim-3) (F38-2E2)	Biolegend	Cat# 345008; RRID:AB_11218598
FITC anti-human CD223 (LAG-3) (11C3C65)	Biolegend	Cat# 369308; RRID:AB_2629751
PE anti-human KLRG1 (MAFA) (SA231A2)	Biolegend	Cat# 367712; RRID:AB_2572157
APC anti-human CD152 (CTLA-4) (BNI3)	Biolegend	Cat# 369612; RRID:AB_2632874
APC-Vio® 770 anti-human CD4 (VIT4)	Miltenyi Biotec	Cat# 130-096-652; RRID:AB_2660925
VioGreen® CD8 anti-human (BW135/80)	Miltenyi Biotec	Cat# 130-096-902; RRID:AB_2660905
VioGreen® CD20 anti-human (REA780)	Miltenyi Biotec	Cat#: 130-111-532; RRID:AB_2656069
VioGreen® CD14 anti-human (Tük4)	Miltenyi Biotec	Cat# 130-096-875; RRID:AB_2660175
FITC CD154 anti-human (5C8)	Miltenyi Biotec	Cat# 130-096-233; RRID:AB_10830229
CD40 Antibody, anti-human, pure-functional grade (HB14)	Miltenyi Biotec	Cat# 130-094-133; RRID:AB_10839704
Alexa Fluor® 488 Anti-CPT1A (8F6AE9)	abcam	Cat# ab171449; RRID:AB_2714024
Anti-IDH2 (EPR7577)	abcam	Cat# ab230796
Anti-Hexokinase 1 (EPR10134(B))	abcam	Cat# ab233837
Anti-Glucose 6 Phosphate Dehydrogenase (EPR6292)	abcam	Cat# ab231828
Anti-Peroxiredoxin 2/PRP (EPR5154)	abcam	Cat# ab227988
PE Anti-Glucose Transporter GLUT1 (EPR3915)	abcam	Cat# ab209449
Alexa Fluor® 700 anti-human CD3 (SK7)	Biolegend	Cat# 344822; RRID:AB_2563420
Brilliant Violet 421™ anti-human CD14 (HCD14)	Biolegend	Cat# 325628; RRID:AB_2563296
Brilliant Violet 510™ anti-human CD19 (HIB19)	Biolegend	Cat# 302242; RRID:AB_2561668
BUV395 Mouse anti-human CD4 (SK3 (Leu3a))	BD Biosciences	Cat# 563552; RRID:AB_2738275
APC-eFluor 780 anti-human CD8a (SK1)	ThermoFisher Scientific	Cat# 47-0087-42; RRID:AB_2016684
Brilliant Violet 785™ anti-human CD56 (NCAM) (5.1H11)	Biolegend	Cat# 362550; RRID:AB_2566059
Brilliant Violet 650™ anti-human CD45RO (UCHL1)	Biolegend	Cat# 304232; RRID:AB_2563462
Brilliant Violet 605™ anti-human CD62L (DREG-56)	Biolegend	Cat# 304834; RRID:AB_2562130

**Chemicals, peptides, and recombinant proteins**		

PepTivator® EBV EBNA-1, human	Miltenyi Biotec	Cat# 130-093-613
PepTivator® EBV LMP2A, human	Miltenyi Biotec	Cat# 130-093-615
PepTivator® EBV BZLF1, human	Miltenyi Biotec	Cat# 130-093-611
Human AB serum	Sigma Aldrich	Cat# H4522
Brefeldin A	Sigma Aldrich	Cat# B5936
Sodium pyruvate	Thermo Fisher Scientific	Cat# BP356-100
L-Glutamine	Thermo Fisher Scientific	Cat# 25030081
Glucose	Sigma Aldrich	Cat# G7528
Methyl tert-butyl ether (MTBE)	Biosolve Chemicals	Cat# 13890602
Derivatization reagents for GC (MSTFA)	Macherey-Nagel	Cat# 701270.510
ULC/MS grade Acetonitrile	Biosolve Chemicals	Cat# 01204102
Acetic acid glacial ULC/MS	Biosolve Chemicals	Cat# 01074131
ULC/MS grade Water	Biosolve Chemicals	Cat# 23214102

**Critical commercial assays**		

Seahorse XF Cell Mito Stress Test Kit	Agilent Technologies	Cat# 103015-100
Seahorse XF Base Medium	Agilent Technologies	Cat# 102353-100
Seahorse Calibrant Solution	Agilent Technologies	Cat# 100840-000
Seahorse XFe96 FluxPak	Agilent Technologies	Cat# 102416-100
RNeasy Plus Mini Kit	Qiagen	Cat# 74134
RevertAid H Minus First Strand cDNA Synthesis Kit	Thermo Fisher Scientific	Cat# K1632
TaqMan® Gene Expression Assay	Thermo Fisher Scientific	Cat# 4331182
CD154 MicroBead Kit, human	Miltenyi Biotec	Cat# 130-092-658
CD3 MicroBeads, human	Miltenyi Biotec	Cat# 130-050-101
CD14 MicroBeads, human	Miltenyi Biotec	Cat# 130-050-201
LS columns	Miltenyi Biotec	Cat# 130-042-401
MS columns	Miltenyi Biotec	Cat# 130-042-201
LIVE/DEAD® Fixable Aqua Dead Cell Stain Kit	Thermo Fisher Scientific	Cat# L34957
Zombie UV™ Fixable Viability Kit	Biolegend	Cat# 423107
Zombie NIR™ Fixable Viability Kit	Biolegend	Cat# 423106
Fixation/Permeablization Kit	BD Biosciences	Cat# 554714; RRID:AB_2869008
eBioscience™ Foxp3 / Transcription Factor Staining Buffer Set	ThermoFisher Scientific	Cat# 00-5523-00
PE/Cy7® Conjugation Kit - Lightning-Link®	abcam	Cat# ab102903
PerCP/Cy5.5® Conjugation Kit - Lightning-Link®	abcam	Cat# ab102911
PE/Atto 594 Conjugation Kit - Lightning-Link®	abcam	Cat# ab269900
Alexa Fluor® 647 Conjugation Kit (Fast) - Lightning-Link®	abcam	Cat# ab269823

**Software and algorithms**		

BD FACSDiva version 6.1.2	BD Biosciences	RRID:SCR_001456
FlowJo 10.1	Treestar Inc., USA	RRID:SCR_008520
GraphPad Prism 7	GraphPad Software Inc., USA	RRID:SCR_002798
Wave Desktop 2.3	Agilent Technologies	RRID:SCR_014526
R Version 4.0.5	R Project for Statistical Computing	RRID:SCR_001905
Leco Chroma-TOF-GC Software v4.50.8.0	LECO	N/A; https://www.leco.com/product/chromatof-software
Python Programming Language 3.8.5	http://www.python.org/	RRID:SCR_008394
MatPlotLib 3.3.1	http://matplotlib.sourceforge.net	RRID:SCR_008624
NumPy 1.19.1	http://www.numpy.org	RRID:SCR_008633
Pandas 1.1.3	https://pandas.pydata.org	RRID:SCR_018214
SciPy 1.5.2	http://www.scipy.org/	RRID:SCR_008058
Seaborn 0.11.0	https://seaborn.pydata.org/	RRID:SCR_018132
Statsmodel 0.12.0	http://www.statsmodels.org/	RRID:SCR_016074

**Other**		

ACQUITY UPLC HSS T3 Column	Waters	Cat# 176001132
ACQUITY UPLC BEH C8 Column	Waters	Cat# 176000885

### Resource availability

#### Lead contact

Requests for further information regarding resources and reagents used in this study should be directed to the lead contact, Prof. Stefan Gold (stefan.gold@charite.de).

#### Materials availability

This study did not generate new unique reagents.

### Experimental model and subject details

#### Participants and clinical assessments

The study was approved by the Ethics Committee of Charité - Universitätsmedizin Berlin (EA1/096/15) and conducted in accordance with relevant international, national and institutional guidelines (Helsinki Declaration of 1975). All participants provided written informed consent and received remuneration for participation. MDD patients, aged 18 to 60 years, were recruited via the in- and outpatient centers of the Department of Psychiatry, Charité - Universitätsmedizin Berlin. HC were recruited via the university hospital's study data bases as well as via print and online advertisements.

Summary and detailed patient characteristics can be found in Tables 1 and S2. MDD patients and HC were matched pairwise for age, sex, smoking status, and BMI to minimize potential confounding effects of these factors on biological variables of interest. Participants were excluded if they had a diagnosis of relevant medical diseases (e.g., diabetes, cardiovascular diseases, autoimmune or infectious illnesses), currently received immunomodulatory drugs (such as non-steroidal anti-inflammatory drugs (NSAIDs), glucocorticoids or antibiotics), or were vaccinated in the last three months. Pregnancy was also an exclusion criterion. For the MDD group, patients had to have a clinician-confirmed diagnosis of MDD and a minimum antidepressant-free period of two weeks. Patients with comorbid psychiatric diagnoses other than mild-to-moderate anxiety disorders were excluded. All HCs did not meet diagnostic criteria for any psychiatric disorder, had a Montgomery Asberg Depression Rating Scale (MADRS) score <7 and no personal or family history of affective disorders (first-degree relatives).

During the eligibility visit, participants underwent a detailed medical history assessment and a thorough physical examination to assess body weight, height, waist/hip ratio, and blood pressure. In addition, blood samples were taken for standard laboratory diagnostics, including: hemoglobin, hematocrit, MCV, MCH, MCHC, RDW-CV, MPV, leukocyte, erythrocyte and platelet counts, differential blood count, CRP, HDL and LDL cholesterol. Clinical diagnosis of MDD according to DSM-5 (American Psychiatric Association and DSM-5 Task Force, 2013) was confirmed by two experienced board-certified psychiatrists (DP, CO) as well as a structured interview (Mini-International Neuropsychiatric Interview = MINI). Clinician rating of depression severity was obtained using the MADRS. MINI and MADRS were conducted by a trained rater (HH). Self-report symptom severity was assessed by questionnaires for anxiety (Beck Anxiety Inventory, BAI), depressive symptoms (Beck Depression Inventory II, BDI-II), and adverse childhood experiences (Childhood Trauma Questionnaire, CTQ).

### Method details

#### Blood collection and isolation of PBMCs

Following an overnight fasting period of 12 h, peripheral blood was collected in heparinized tubes (BD, Germany) between 8.00 a.m. and 9.30 a.m. PBMCs were isolated within 1 h of collection via density gradient centrifugation. In short, diluted blood [1:1 with phosphate-buffered saline (PBS, Gibco, ThermoFisher Scientific, Germany)] was layered on density medium (Biocoll, Biochrome, Germany), centrifuged, and the PBMC layer obtained. PBMCs were washed twice in PBS, counted, and resuspended in RPMI-1640 + GlutaMax medium (Gibco, ThermoFisher Scientific) supplemented with 25% heat-inactivated fetal calf serum (FCS) (Biochrome) and 10% dimethylsulfoxide (DMSO) (Applichem GmbH, Germany) at 10^7^ cells/ml for cryopreservation in liquid nitrogen. Cryopreserved PBMCs were thawed in a 37°C water bath for 2 min before being transferred into 10 ml of 37°C thawing medium (RPMI-1640 + GlutaMax + 10% FCS). For antigen-reactivity experiments, FCS was replaced with 5% human AB serum (Sigma-Aldrich, Germany). Cells were then washed with medium, counted and prepared for subsequent experiments as described below.

#### Blood cholesterol measurements

Serum high density lipoprotein (HDL) and low-density lipoprotein (LDL) cholesterol levels were determined by a clinically licensed diagnostic lab (Labor Berlin - Charité Vivantes GmbH, Germany).

#### Metabolomics and lipidomics

*General sample preparation and extraction for LC- and GC-MS:* The sample preparation was performed according to MetaSysX standard procedure, a modified protocol from Salem et al. (2016). Briefly, 50 μl of serum from 22 MDD patients and corresponding matched HC was extracted by methyl-tert-butyl-ether (MTBE)/methanol/water solvent system that separates molecules into aqueous and organic phase respectively. After MTBE extraction fractions were collected to new tubes. Additional 150 μl of polar phase was transferred to a tube for subsequent derivatization and GC-MS analysis. All the samples in new tubes were dried down using a centrifugal evaporator and stored at −80°C until LC-MS and GC-MS analysis.

##### Liquid phase chromatography and mass spectrometry (LC-MS) sample preparation

The dried samples were resuspended in 100 μl of water or acetonitrile for polar and lipid measurements respectively.

##### LC-MS measurements

The samples were measured with a Waters ACQUITY Reversed Phase Ultra Performance Liquid Chromatography (RP-UPLC) coupled to a Thermo-Fisher Exactive Plus mass spectrometer (Thermo Fisher Scientific). C8 (100 mm × 2.1 mm × 1.7μm particles; Waters) and C18 (100 mm × 2.1 mm × 1.8μm particles; Waters) columns were used for the lipophilic and the hydrophilic measurements, respectively. A 15 min gradient was used for separation of polar and lipophilic compounds. The mobile phases for separation of polar and semi-polar compounds were 0.1% formic acid in H_2_O (buffer A) and 0.1% formic acid in acetonitrile (buffer B). The chromatographic separation of these analytes was performed in the following conditions: A 99% initial to 1 min, A 99% to A 60% to 11 min, A 60% to A 30% to 13 min and A30% to A 1% to 15 min. The following mobile phases were used for lipids and lipophilic metabolites separation: 1% of 1M NH4Ac in 0.1% acetic acid (buffer A) and acetonitrile: isopropanol (7:3) containing 1% of 1M NH4Ac in 0.1% acetic acid (buffer B). The separation of lipids and lipophilic compounds was performed with a step gradient from 45% initial to 1 min, 45% A to 25% A in 4 min, 25%–11% A in 11 min and 11%–0% A in 15 min. Chromatograms were recorded in Full Scan MS mode (Mass Range [100–1500]). All mass spectra were acquired in positive and negative mode.

##### LC-MS data processing and annotation

LC-MS RAW data files were extracted with the PeakShaper -metaSysX GmbH internal software. Alignment and filtering of the LC-MS data were completed using in-house software. An in-house metaSysX database of chemical compounds was used to match the features detected in the LC-MS polar and non-polar platform. The metaSysX database contains the mass-to-charge ratio and the retention time information of reference compounds measured at the same chromatographic and spectrometric condition as samples measurements. A 5 ppm and 0.1 min deviation from the reference compounds mass-to-charge-ratio and retention time respectively were used as matching criteria.

##### Gas chromatography and mass spectrometry (GC-MS) sample preparation

The derivatisation of the metabolites for GC-MS analysis was performed according to previously described method (Lisec et al., 2006). Briefly, the dried down samples were suspended in methoxy-hydrochlorid/pyridine solution and incubated at 30°C for 90 min followed by derivatization with N-methyl-N-trimethylsilyltrifluoroacetamide (MSTFA) at 37°C for 30 min.

##### GC-MS measurements

The samples were measured on an Agilent Technologies GC coupled to a Leco Pegasus HT mass spectrometer which consists of an EI ionization source and a TOF mass analyzer. Column: 30 m DB35; starting temp: 85°C for 2 min; gradient: 15°C per min up to 360°C.

##### GC-MS data processing and annotation

NetCDF files that were exported from the Leco Pegasus software were imported into the “R” Bioconductor package TargetSearch (Cuadros-Inostroza et al., 2009) to transform retention time to retention index (RI), to align the chromatograms, to extract the peaks, and to annotate them by comparing the spectra and the RI to the Fiehn Library and to a user created library. Annotation of peaks was manually confirmed in Leco Pegasus. Analytes were quantified using a unique mass. Metabolites with a RT and a mass spectrum that did not have a match in the database were labeled as unknown.

##### Data analysis

Feature intensities were log2-transformed and platform-wise subjected to measurement-day- and sample-median-normalization as well as filtering (retention time, group-wise mean intensity). Features with more than 20% missing values were discarded, the final dataset exhibited a total of 3511 features. Differential expression of features with respect to healthy and depressed groups was assessed by means of a linear model. We added age, body mass index, sex, measurement day, smoking status and thawing cycle as predictors to control for potential confounding. We controlled the false-discovery rate for multiple comparisons platform-wise by the procedure of Benjamini & Hochberg (Benjamini and Hochberg, 1995). Features were considered differentially expressed if the FDR-corrected p values with respect to study groups was below α = 0.05. Associations of features with endpoints were quantified via partial correlations. To this end, we predicted feature intensities and endpoints by a linear model comprising the potential confounders age, body mass index, sex, measurement day, smoking status and thawing cycle. We calculated Spearman's rank correlation coefficient from the residuals. False-discovery-rate was platform-wise controlled for multiple comparisons by the procedure of Benjamini & Hochberg. Associations were considered significant if the FDR-corrected p values were below α = 0.05. Due to limited availability of biomaterial from study participants BHC050, BHC053 BMDD010, BMDD013, BMDD023 and BMDD031 these subjects were not part of the statistical analysis.

#### Purification of T cells and monocytes

After thawing of cryopreserved PBMCs, CD3^+^ T cells and CD14^+^ monocytes were purified sequentially via magnetic-activated cell sorting (MACS, Miltenyi Biotec, Germany). In brief, PBMCs were incubated with 20 μl of α-CD3 Microbeads (Miltenyi Biotec) and 80 μl of MACS buffer (0.5% BSA in AutoMACS rinsing solution, both Miltenyi Biotec) per 10^7^ cells for 15 min at 4°C, washed and taken up in MACS buffer. Labeled cells were then applied to an LS column (Miltenyi Biotec) for CD3^+^ T cell positive selection according to the manufacturer's instructions. Subsequently, CD14^+^ monocytes were purified from the negative fraction with α-CD14 Microbeads (Miltenyi Biotec) in an analogous manner. In our hands, this procedure yields a cell purity of 96.5 + 1.2% for T cells and 92.3 + 1.7% for monocytes.

#### Cellular respiration assay

Live cell metabolic assessments were conducted using the Cell Mito Stress Assay in a Seahorse XF^e^ 96 Analyzer, optimized for use with cryopreserved primary human immune cells as described (Taenzer, 2019). Briefly, magnetically purified CD3^+^ T cells and CD14^+^ monocytes were rested for 2 h at 37°C and 5% CO_2_ in RPMI1640 medium + GlutaMAX +10% FCS at a density of 2 × 10^6^ cells/ml. Following a washing step with Seahorse XF Base Medium (Agilent Technologies, USA) supplemented with 1 mM sodium pyruvate, 2 mM L-glutamine (both ThermoFisher Scientific), 10 mM glucose (Sigma Aldrich) and adjusted to pH 7.4. Cells were then transferred to a 96-well Seahorse cell culture plate in Seahorse medium at a density of 4 × 10^5^ cell per well in 3-5 replicates. The plate was incubated for 30 min at 37°C in a non-CO_2_ environment. The sensor cartridge was pre-hydrated with calibration buffer (Agilent Technologies) for 4 h and the injection ports were filled with oligomycin, FCCP, rotenone/antimycin A at final assay concentrations of 2 μM, 1 μM or 0.5 μM, respectively (Seahorse XF Cell Mito Stress Test Kit, Agilent Technologies), or Seahorse medium. After an automated calibration process according to the manufacturer's instructions the assay plate was measured in a Seahorse XF^e^ 96 Analyzer (Agilent Technologies). Key metabolic parameters were analyzed with Wave software (Agilent Technologies) (Figure S1). Matched patient/HC pairs were measured on the same plate to avoid day-to-day variations between pairs.

#### Analysis of T cell phenotype

Antibody panels for flow cytometric analysis of T cell phenotype are depicted in Table S1. Thawed PBMCs were incubated with a live/dead marker (Zombie NIR Fixable Viability Kit, Biolegend, UK) in PBS for 15 min (together with the CCR7 antibody for the naive/memory T cell panel). Antibody premixes (see Table S1, all Biolegend) were prepared in staining buffer [PBS +0.5% bovine serum albumin (Miltenyi Biotec) + 2 mM EDTA (Promega, USA) + 0.02% sodium azide (Sigma-Aldrich)] and added to the cells for further 15 min. Following a washing step, samples were resuspended in staining buffer and measured on a FACSCanto II flow cytometer (BD, Germany). All flow cytometry data described (below) were analyzed using FlowJo version 10.1 software (Treestar Inc., USA).

#### Analysis of inhibitory receptors and KLRG1

After thawing of PBMCs, each 5 × 10^5^ cells were seeded into two wells of a 96-well round bottom plate and rested over night at 37°C, 5% CO_2_. The next day, the cells were stimulated with 10 μg/ml of α-CD3 antibody and 1 μg/ml α-CD28 antibody or left unstimulated for 48 hours. Subsequently, PBMCs were harvested, washed with PBS and stained with the respective staining mix (Table S1) for 15 min at room temperature. Cells were washed and resuspended in staining buffer before flow cytometric analysis.

#### Analysis of EBV-reactive T cells

3-10 × 10^6^ PBMCs were seeded at a density of 5 × 10^6^ cells/cm^2^ in cell culture plates in RPMI1640 medium + GlutaMAX +5% human AB serum and incubated over night at 37°C, 5% CO_2_. The next morning, cells were stimulated with EBV antigens (EBNA-1 + LMP2a + BZLF1 peptide pools), MP65 peptide pool (all Miltenyi Biotec, final concentration 0.6 nmol/ml/peptide) or left unstimulated for 7 h. To avoid downregulation of the CD154 activation marker, α-CD40 antibody was added during the incubation period at 1 μg/ml (Miltenyi Biotec). After 5 hours 2 μg/ml Brefeldin A (Sigma-Aldrich) was added for the last two hours of stimulation. The cells were harvested and a small aliquot was removed for later FACS staining (= original fraction; for all antibody panels see Table S1). PBMCs were washed with cold staining buffer and labeled with α-CD154 Biotin + α-Biotin Microbeads according to the manufacturer’s instruction (CD154 MicroBead Kit, Miltenyi Biotec) with a slight adaptation: The α-Biotin-antibody was added in combination with the surface antibody mix in PBS. After a washing step the cells were fixed with fixation/permeabilization solution (BD) and transferred to an equilibrated MS separation column attached to a strong magnet (Miltenyi Biotec). The column was rinsed twice with Perm/Wash buffer (BD) and the intracellular staining mix was added directly onto the column in Perm/Wash buffer. After a 12 min incubation time, the column was washed again with Perm/Wash buffer and the enriched cells were eluted with staining buffer (= CD154 enriched fraction). The CD154 enriched fraction was measured on a FACS Canto II flow cytometer (BD). For analysis, the number of non-specifically activated background cells was subtracted from the positive signal. The frequency of antigen-reactive T cells was calculated by dividing the absolute number of CD4^+^/CD154^+^ T cells after enrichment by the absolute number of CD4^+^ T cells within total PBMCs before enrichment.

#### Cell-specific gene expression by qPCR

CD3^+^ T cells and CD14^+^ monocytes were isolated from thawed PBMCs by magnetic-activated cell sorting, as described above. RNA isolation was performed using Qiagen RNeasy Plus Mini Kit (Qiagen, Germany) according to manufacturer's instructions. Concentration and purity were measured with a NanoDrop spectrophotometer (NanoDrop 2000c, ThermoFisher Scientific). RNA was then transcribed to complementary DNA (cDNA) using the RevertAid H Minus First Strand cDNA Synthesis Kit (ThermoFisher Scientific), according to manufacturer's instructions. cDNA amplification was carried out on a StepOne Real-Time PCR system (Applied Biosystems, Germany) using TaqMan Gene Expression Assays (ThermoFisher Scientific) for *TNF* (Hs01113624_g1), *SLC2A1* (Hs00892681_m1) (encoding GLUT1) and *CPT1a* (Hs00912671_m1). All RT-qPCR reactions were performed in triplicates with matched control and patient samples always on the same plate. Results are given as relative gene expression (normalized to the geometric mean of the two housekeeping genes, TATA Box Binding Protein (*TBP*; HS00427620_m1) and Importin 8 (*IPO8*; Hs00183533_m1).

#### Met-flow

Met-Flow, a flow cytometry-based method capturing the metabolic state of immune cells by targeting key proteins and rate-limiting enzymes across multiple pathways (Ahl et al., 2020), was used in a subset of n = 5 MDD cases and n = 5 matched healthy controls. The antibody panel used for flow cytometry-based metabolic readouts is provided in Table S1. The key metabolic proteins CPT1a, GLUT1, PRDX2, G6PD, IDH2 and HK1 were chosen for analysis.

PBMCs were thawed and 1.5 × 10^6^ cells were seeded into round bottom 96 well plates at a density of 0.5 × 10^6^ cells/well in RPMI1640 medium +10% FCS and incubated for 2 hrs at 37°C, 5% CO_2_. Cells were then pre-stained with a Live/Dead marker (Zombie UV Fixable Viability Kit, Biolegend) in PBS for 20 min at RT in the dark. Following a washing step with FACS buffer (PBS + 2 % BSA), surface staining antibody mix prepared in PBS was added to the cells and incubated for 30 min at RT protected from light. After a washing step (Perm/wash solution, Invitrogen eBioscience Foxp3/Transcription Factor Staining Buffer Set, Thermo Fisher Scientific), cells were fixed with fixation/permeabilization solution (Invitrogen eBioscience Foxp3/Transcription Factor Staining Buffer Set, Thermo Fisher Scientific) for 30 min, followed by intracellular staining in permeabilization buffer for 30 min at RT in the dark. PBMCs were washed once more with Perm/wash solution, resuspended in FACS buffer and measured on a FACS Fortessa flow cytometer (BD).

### Quantification and statistical analysis

#### Statistical analysis

Continuous variables were analyzed with Wilcoxon signed-rank test based on the pairwise matching of MDD patients and HC (for age, sex, smoking, and BMI). Dichotomous variables were analyzed with McNemar's test.

Planned and pre-specified primary analyses used the standard, unadjusted alpha level of 0.05 for each variable of interest (each systemic metabolic marker, each cellular respiration read-out, and all cell subset frequencies obtained from immunophenotyping). In order to corroborate these results, we also conducted secondary analyses adjusting for multiple comparisons per group of variables of interest using Bonferroni adjustments (i.e. an alpha level of 0.0125 for the four systemic markers LDL/HDL ratio, WHR, systolic and diastolic blood pressure, an alpha level of 0.0071 for the seven parameters of cellular respiration in T cells and monocytes, and an alpha level of 0.005 for ten MADRS items, respectively). Gene expression analyses were considered exploratory and not adjusted for multiple comparisons. Statistical details (statistical tests used, value of n, definition of center value as well as dispersion and precision measures) can also be found in the respective figure legends.

Data management was performed with Excel (Microsoft, USA) and statistical analyses were conducted with GraphPad Prism version 7 (GraphPad Software Inc., USA). Spearman rank correlation between systemic and T cell markers with MADRS items, as well as correlation of metabolic markers with CD3 and CD14 seahorse readouts was done using R (version 4.0.5). Statistical analyses of the metabolomics and lipidomics data were performed using Python 3.8.5 with the following modules: NumPy 1.19.1, Pandas 1.1.3, SciPy 1.5.2 and statsmodels 0.12.0. The volcano plot was created with Python 3.8.5 and MatPlotLib 3.3.1.

## Data Availability

• Data on cellular metabolism measurements are available as a supplementary file (Data S1). Further data reported in this paper will be shared by the lead contact upon request. • No custom code was used in the analysis of the data. • Any additional information required to reanalyze the data reported in this paper is available from the lead contact upon request.

## Supplemental information

Document S1. Figures S1–S5 and Tables S1, S2

Data S1. Individual patient data for Seahorse assay readouts, related to Figures 2 and S2
