# Supplementary material for: Reduced mitochondrial respiration in T cells of patients with major depressive disorder
Source: iScience. 2021 Oct 16;24(11):103312. doi: 10.1016/j.isci.2021.103312 (PMC8571492; doi:10.1016/j.isci.2021.103312)
Supplement: Document S1. Figures S1–S5 and Tables S1, S2 [file mmc1.pdf]

iScience, Volume *24*

Supplemental information

Reduced mitochondrial respiration in T cells of patients with major depressive disorder

Stefanie Gamradt, Helge Hasselmann, Aline Taenzer, Jelena Brasanac, Victoria Stiglbauer, Arne Sattler, Max Sajitz-Hermstein, Sylwia Kierszniowska, Caren Ramien, Jan Nowacki, Lea Mascarell-Maricic, Katja Wingenfeld, Dominique Piber, Andreas Ströhle, Katja Kotsch, Friedemann Paul, Christian Otte, and Stefan M. Gold

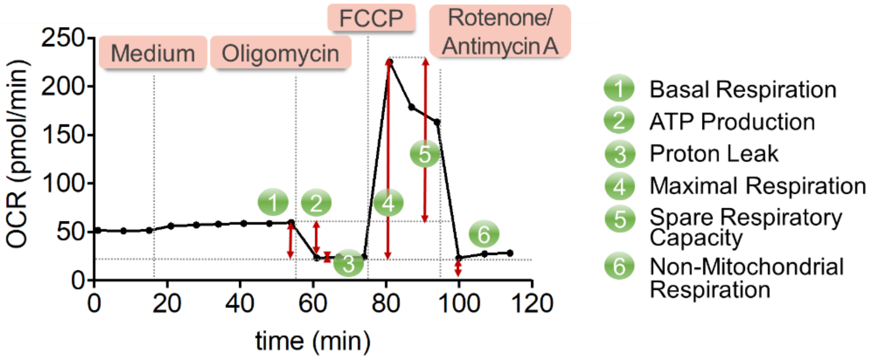

Figure S1 | Key parameters of mitochondrial respiration derived from oxygen consumption rate (OCR) measurements using Seahorse XF Cell Mito Stress Test (Related to Figure 2, Figure S2 and STAR Methods). In order to quantify mitochondrial respiration, the following modulators of respiration were added sequentially to the cells and the OCR was determined over time in a Seahorse XFe96 Analyzer: Oligomycin as ATP synthase inhibitor, carbonyl cyanide-4(trifluoromethoxy) phenylhydrazone (FCCP) as uncoupling agent and Rotenone/Antimycin A as complex I/III inhibitors. At the beginning of the assay fresh Seahorse medium was added.

Calculations:
Basal Respiration = Last rate measurement before addition of Oligomycin – Non-Mitochondrial Respiration
ATP Production = Last rate measurement before Oligomycin injection – Minimum rate measurement after Oligomycin injection
Proton Leak = Minimum rate measurement after Oligomycin injection – Non-Mitochondrial Respiration
Maximal Respiration = Maximum rate measurement after FCCP injection – Non-Mitochondrial Respiration
Spare Respiratory Capacity = Maximal Respiration – Basal Respiration
Non-mitochondrial Respiration = Minimum rate measurement after Rotenone/ Antimycin A injection
Coupling Efficiency (%) = ATP Production / Basal Respiration x 100 (values >100% were interpreted as 100%)

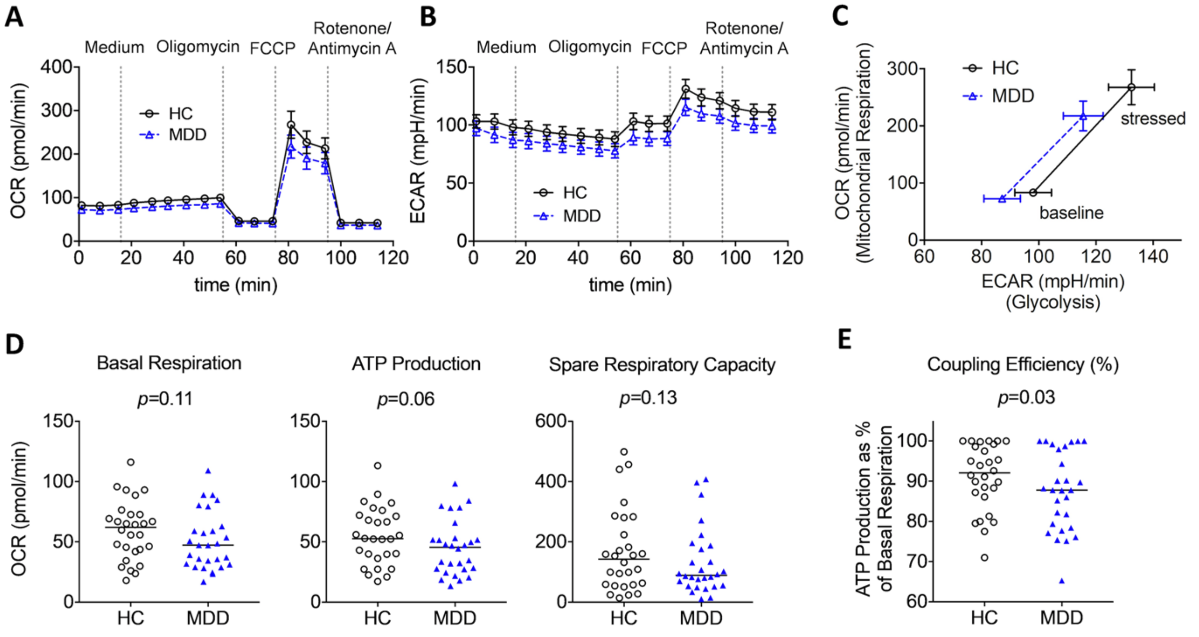

Figure S2 | Immunometabolic profiling of monocytes from MDD patients (Related to Figure 2). In order to quantify mitochondrial respiration as well as glycolytic activity of $CD14^+$ monocytes from 28 MDD patients and matched healthy controls (HC), different modulators of respiration were added sequentially to the cells and the oxygen consumption rate (OCR) as well as the extracellular acidification rate (ECAR) were determined over time in a Seahorse XFe96 Analyzer (mean ± SEM is shown, all assays run in three to five replicates for each subject[1]) (A, B). OCR and ECAR values under baseline and stressed conditions (= after addition of Oligomycin and FCCP) were plotted against each other to visualize the energy phenotype of the cells; mean ± SEM is shown (C). The main parameters of mitochondrial respiration (basal respiration, ATP production and spare respiratory capacity) as well as the coupling efficiency expressed as the % of basal respiration coupled to ATP production are shown (D, E). See also **Figure S1** for further information regarding the calculation of these parameters from OCR measurements. Data are presented as individual data and group median. All cells were freshly isolated on the day of the patient visit, then cryopreserved and defrosted on the day of experiment. Wilcoxon signed-rank test was used to compare groups. Oligomycin: ATP synthase inhibitor; carbonyl cyanide-4(trifluoromethoxy) phenylhydrazone (FCCP): uncoupling agent; Rotenone/Antimycin A: complex I/III inhibitors. Individual patient data for the readouts presented here are provided in the supplementary data file, **Data S1.**

[1] Assay run in duplicates for subjects BHC22, BHC119, BHC120, BMDD2, BMDD8 and BMDD11

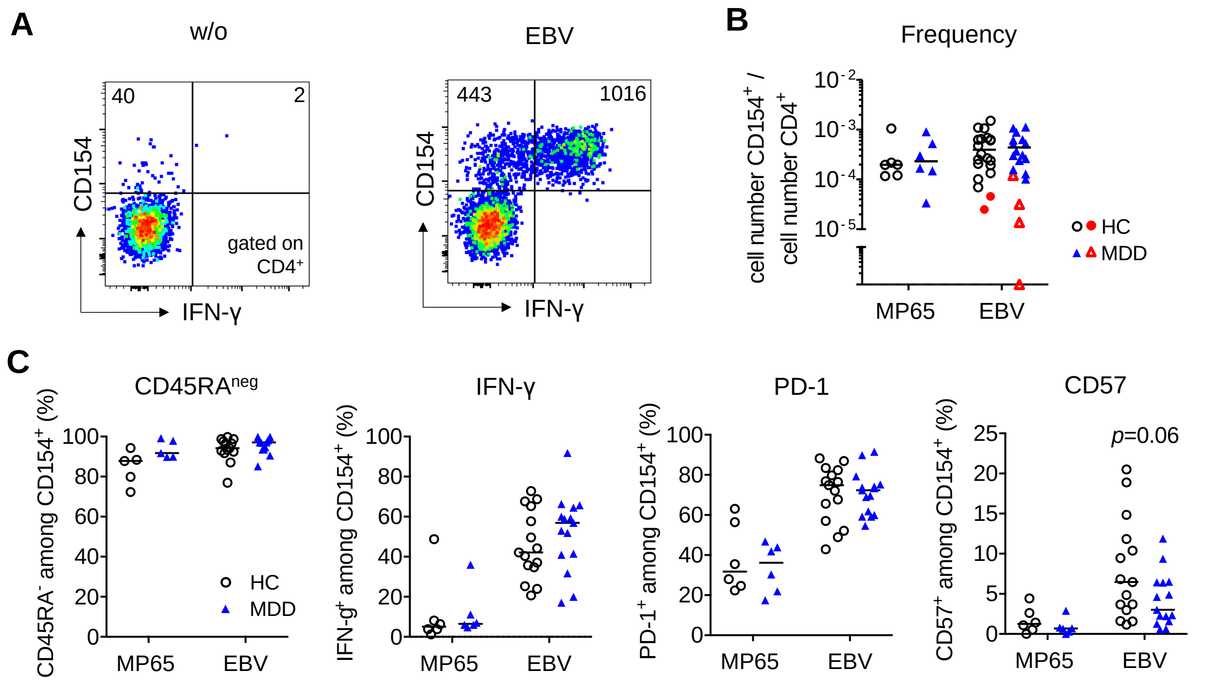

Figure S3 | Characterization of EBV-specific T cells in MDD patients (Related to Figure 3). Cryopreserved and thawed PBMCs from MDD patients and matched healthy controls (HC) were stimulated with a pool of EBV antigens (EBNA-1, LMP2a, BZLF; n=20 pairs), the control antigen MP65 (n=6 pairs) from *C. albicans* or left unstimulated for 7 h. Samples were then magnetically enriched for antigen-activated $CD154^+$ T cells and analyzed by flow cytometry. Exemplary dot plots of a non-stimulated and an EBV-stimulated sample from a HC are shown, numbers indicating absolute cell counts after enrichment from 10^7 PBMCs (A). Relative frequencies of EBV-reactive T cells in MDD patients and HC (B). Red filled circles (HC) or open triangles (MDD) indicate EBV-negative individuals which could be clearly identified by high CD45RA expression and low expression of IFN- γ (not shown). Expression levels of CD45RA, IFN-γ, PD-1 and CD57 in EBV-specific T cells of MDD patients and HC are shown (C). Individual patient data and median are shown. Wilcoxon signed-rank test was used to compare groups. All p-values > 0.1 if not otherwise indicated.

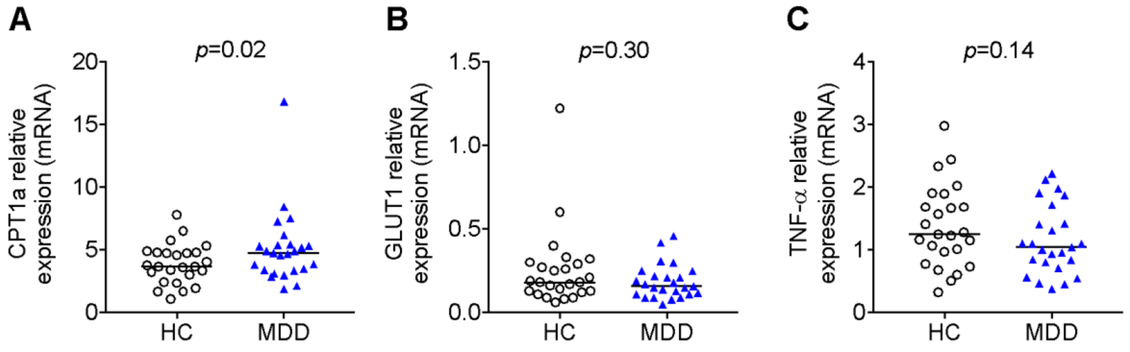

Figure S4 | Expression of key regulator genes of cellular metabolism in monocytes of MDD patients (Related to Figure 4). mRNA expression levels of *CPT1a* (A), *SLC2A1* (encoding GLUT1) (B) and *TNF* (C) in $CD14^{+}$ monocytes of MDD patients (n=25) compared to matched controls (HC, n=25). Gene expression is shown as fold change relative to housekeeping genes (*TBP* and *IPO8*). Individual patient data and median are shown. Wilcoxon signed-rank test was used to compare groups.

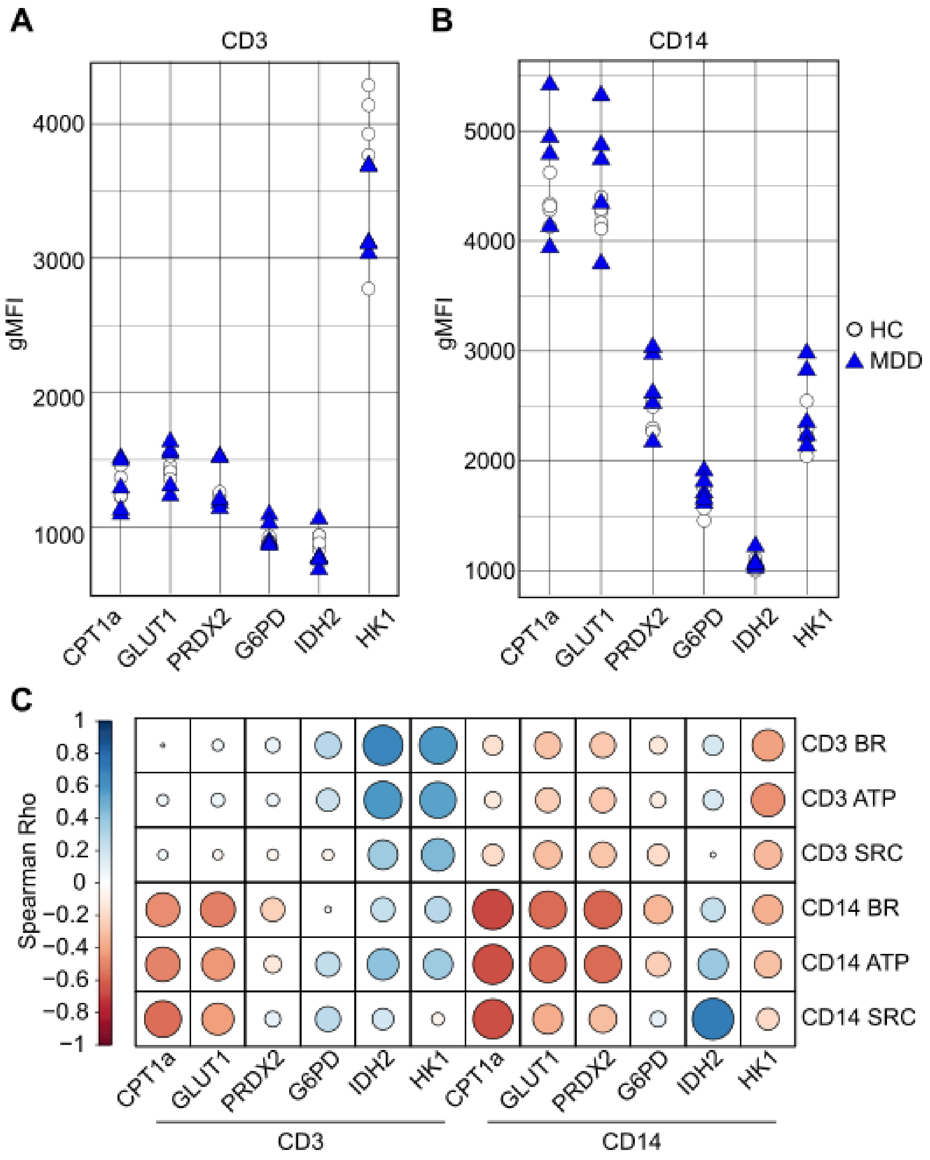

Figure S5 | Flow cytometry-based metabolic analysis of T cells and monocytes of MDD patients (n=5) and healthy controls (HC, n=5) (Related to Figure 2 & 4 and Figure S2 & S4). Geometric mean fluorescence intensities (gMFI) of CPT1a, G6PD, GLUT1, HK1, IDH2 and PRDX2 are shown for $CD3^{+}$ T cells (A) and $CD14^{+}$ monocytes (B). Correlation analyses of Met-Flow readouts of $CD3^{+}$ and $CD14^{+}$ cells with Seahorse assay readouts of T cells and monocytes (C). Circle sizes indicate magnitude of correlation, the color shading additionally depicts the direction of correlation (Spearman's rho, see legend). CPT1a: Carnitine Palmitoyltransferase 1 A; G6PD: Glucose-6-phosphate-dehydrogenase; GLUT1: Glucose transporter 1; HK1: Hexokinase 1; IDH2: Isocitrate dehydrogenase 2; PRDX2: Peroxiredoxin 2; BR: Basal respiration; ATP: ATP production; SRC: Spare respiratory capacity.

Table S1 | Antibody Panels (Related to STAR Methods).

	Target / Fluorochrome	Clone	Company
**Naive/ Memory T cells**	CD3 Brilliant Violet 421	OKT3	Biolegend
	CD8 Brilliant Violet 510	SK1	Biolegend
	HLA-DR FITC	LN3	Biolegend
	CCR7 PE	G043H7	Biolegend
	CD4 PerCP Cy5.5	RPA-T4	Biolegend
	CD45RA PE Cy7	HI100	Biolegend
	CD38 APC	HB-7	Biolegend
	Zombie NIR (live/dead)		Biolegend
**T cell subsets/ CCR**	CD3 Brilliant Violet 421	OKT3	Biolegend
	CD8 Brilliant Violet 510	SK1	Biolegend
	HLA-DR FITC	LN3	Biolegend
	CXCR3 PE	G025H7	Biolegend
	CD4 PerCP Cy5.5	RPA-T4	Biolegend
	CCR6 PE Cy7	G034E3	Biolegend
	CCR4 APC	L291H4	Biolegend
	Zombie NIR (live/dead)		Biolegend
**Tregs**	FoxP3 Brilliant Violet 421	206D	Biolegend
	CD3 Brilliant Violet 510	SK7	Biolegend
	HLA-DR FITC	LN3	Biolegend
	CD25 PE	M-A251	Biolegend
	CD4 PerCP Cy5.5	RPA-T4	Biolegend
	CD45 RA PE Cy7	HI100	Biolegend
	CD127 APC	A019D5	Biolegend
	Zombie NIR (live/dead)		Biolegend
**CD154 enriched fraction**	CXCR5 Brilliant Violet 421	J252D4	Biolegend
	CD8/CD20/ CD14 VioGreen	BW135/80 /REA780/Tük4	Miltenyi Biotec
	Live/Dead Aqua		Thermo Fisher
	CD154 FITC[a]	5C8	Miltenyi Biotec
	PD-1 PE	EH12.2H7	Biolegend
	IFN-γ PerCP-Cy5.5[a]	4S.B3	Biolegend
	CD45RA PE/Cy7	HI100	Biolegend
	CD57 APC	HNK-1	Biolegend
	CD4 APCVio770	VIT4	Miltenyi Biotec

**Senescence / exhaustion (original fraction)**	CXCR5 Brilliant Violet 421	J252D4	Biolegend
	CD8/CD20/ CD14 VioGreen	BW135/80 /REA780/Tük4	Miltenyi Biotec
	Live/Dead Aqua		Thermo Fisher
	CPT1a AF488 or Isotype	8F6AE9/7E10G10	abcam
	PD-1 PE	EH12.2H7	Biolegend
	CD4 PerCP-Cy5.5	RPA-T4	Biolegend
	CD45RA PE/Cy7	HI100	Biolegend
	CD57 APC	HNK-1	Biolegend
	CD8a APC/Cy7	RPA-T8	Biolegend
**Inhibitory receptors/KLRG1**	Tim-3 Brilliant Violet 421	F38-2E2	Biolegend
	CD14/CD20 VioGreen	Tük4/REA780	Miltenyi Biotec
	Live/Dead Aqua		Thermo Fisher
	LAG-3 FITC	11C3C65	Biolegend
	KLRG1 PE	SA231A2	Biolegend
	CD4 PerCP-Cy5.5	RPA-T4	Biolegend
	CD45RA PE/Cy7	HI100	Biolegend
	CTLA-4 APC	BNI3	Biolegend
	CD8 APC/Cy7	RPA-T8	Biolegend
**Met-Flow**	IDH2 PE/Cy7[b]	EPR7577	abcam
	HK1 AF647[b]	EPR10134(B)	abcam
	G6PD PerCPCy5.5[b]	EPR6292	abcam
	PRDX2 Pe-Atto594[b]	EPR5154	abcam
	CPT1a AF488	8F6AE9	abcam
	GLUT1 PE	EPR3915	abcam
	CD14 BV421	HCD14	Biolegend
	CD3 Alexa700	SK7	Biolegend
	CD4 BUV395	SK3 (Leu3a)	BD Biosciences
	CD8a APC-eFluor780	SK1	ThermoFisher Scientific
	CD19 BV510	HIB19	Biolegend
	CD56 BV785	5.1H11	Biolegend
	CD45RO BV650	UCHL1	Biolegend
	CD62L BV605	DREG-56	Biolegend
	Live/Dead UV		Biolegend

TABLE S1 | continued

[a]Antibody conjugates in italic were used for intracellular staining.

[b]Antibodies were custom-conjugated according to manufacturer’s instructions using abcam Lightning Link conjugation kits.

Table S2 | List of patients with MDD and healthy controls matched for age, sex, body mass index and current smoking status (Related to Table 1). Depression severity (MADRS), psychiatric comorbidity as well as depression subtype according to DSM-5 are given where applicable.

	Age	Sex	BMI	Currently Smoking	MADRS Score	Psychiatric Comobidity	Subtype (DSM-5)
MDD017	19	F	21.6	Y	19	-	-
HC065	19	F	21.6	Y	0		
MDD026	30	M	26.5	Y	28	SP	melancholic
HC053	27	M	25.3	Y	0		
MDD030	47	F	19.5	N	27	GAD	melancholic
HC028	42	F	19.0	N	0		
MDD025	32	M	24.3	N	19	PD	melancholic
HC027	31	M	27.5	N	0		
MDD013	26	M	22.2	N	21	-	-
HC038	28	M	22.2	N	1		
MDD012	53	F	27.0	N	28	-	-
HC026	55	F	25.4	N	1		
MDD028	24	F	26.1	Y	25	-	melancholic
HC046	30	F	25.8	Y	1		
MDD031	26	M	18.3	Y	32	-	melancholic
HC077	23	M	17.7	Y	5		
MDD036	24	F	28.7	N	25	-	melancholic
HC024	28	F	26.6	N	0		
MDD023	35	F	19.7	Y	22	-	melancholic
HC045	30	F	19.1	Y	0		
MDD029	39	F	20.3	N	28	-	-
HC022	41	F	23.1	N	0		
MDD027	59	M	25.4	N	25	-	melancholic
HC050	57	M	21.6	N	0		
MDD035	18	F	30.4	N	19	SP	-
HC096	21	F	32.1	N	2		
MDD034	24	M	25.3	N	12	-	-
HC097	30	M	23.0	N	0		

	Age	Sex	BMI	Currently Smoking	MADRS Score	Psychiatric Comorbidity	Subtype (DSM-5)
MDD004	22	F	21.2	N	27	SP	-
HC030	25	F	19.5	N	2		
MDD011	30	F	27.7	N	28	PD	-
HC033	25	F	23.7	N	2		
MDD010	50	F	26.7	N	27	-	melancholic
HC003	44	F	28.5	N	0		
MDD005	20	F	26.6	N	30	-	melancholic
HC034	24	F	22.2	N	0		
MDD006	45	F	22.0	Y	25	-	melancholic
HC071	52	F	22.5	Y	1		
MDD020	29	F	21.9	N	35	-	melancholic
HC106	26	F	24.8	N	5		
MDD038	25	F	21.0	N	31	PTSD	atypical
HC093	30	F	24.1	N	0		
MDD016	36	M	27.3	N	24	PD	melancholic
HC070	32	M	25.7	N	1		
MDD008	55	F	32.2	N	24	-	melancholic
HC119	46	F	31.9	N	0		
MDD032	27	F	25.3	Y	31	SP	-
HC120	36	F	24.6	Y	1		
MDD002	32	F	27.7	N	26	PD	melancholic
HC029	28	F	23.5	N	1		
MDD018	33	F	24.4	N	25	-	melancholic
HC036	28	F	22.4	N	0		
MDD039	20	F	23.7	N	13	-	-
HC044	21	F	25.1	N	2		
MDD040	21	F	22.9	N	32	-	melancholic
HC004	22	F	21.9	N	1		

Table S2 | continued

F: Female; M: Male; Y: Yes; N: No; GAD: generalized anxiety disorder; PD: panic disorder; PTSD: post-traumatic stress disorder; SP: social phobia; MADRS: Montgomery-Asberg Depression Rating Scale; DSM-5: Diagnostic and Statistical Manual of Mental Disorders.
